# 2-[(2*Z*)-Azepan-2-yl­idene]-1-(4-nitro­phen­yl)ethanone

**DOI:** 10.1107/S1600536812032035

**Published:** 2012-07-18

**Authors:** Siyanda T. Mthembu, Lee G. Madeley, Charles B. de Koning, Joseph P. Michael

**Affiliations:** a Molecular Sciences Institute, School of Chemistry, University of the Witwatersrand, Johannesburg, PO Wits 2050, South Africa

## Abstract

The title compound, C_14_H_16_N_2_O_3_, is an NH-vinyl­ogous amide (enaminone) produced by the reaction of 4-nitro­phenacyl bromide with azepane-2-thione. The conformation about the C=C bond [1.3927 (14) Å] is *Z*, which allows for the formation of an intra­molecular N—H⋯O hydrogen bond that leads to an *S*(6) loop. Inversion-related mol­ecules associate *via* N—H⋯O hydrogen bonds to form a 12-membered {⋯OC_3_NH}_2_ synthon.

## Related literature

For uses and reactions of enamino­nes, see: Roth *et al.* (1971 ▶); Paulvannan & Stille (1994 ▶); Michael *et al.* (1999 ▶). For related structures, see: Balderson *et al.* (2007 ▶). For graph-set notation, see: Bernstein *et al.* (1995 ▶).
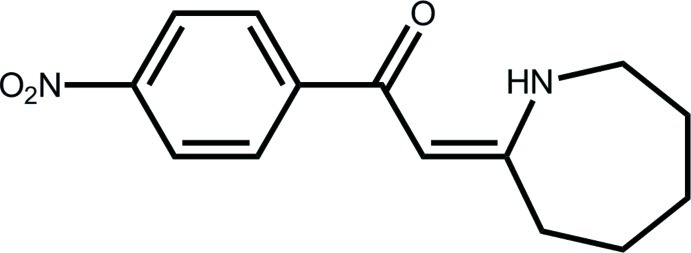


## Experimental

### 

#### Crystal data

C_14_H_16_N_2_O_3_
*M* *_r_* = 260.29
Triclinic, 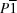

*a* = 6.7963 (3) Å
*b* = 8.4054 (3) Å
*c* = 11.6649 (5) Å
α = 76.508 (2)°
β = 81.134 (2)°
γ = 80.596 (2)°
*V* = 634.58 (5) Å^3^
*Z* = 2
Mo *K*α radiation
μ = 0.10 mm^−1^
*T* = 173 K
0.56 × 0.5 × 0.42 mm

#### Data collection

Bruker APEXII CCD area-detector diffractometer
Absorption correction: multi-scan (*SADABS*; Sheldrick, 1996 ▶) *T*_min_ = 0.905, *T*_max_ = 0.955
10354 measured reflections
3041 independent reflections
2732 reflections with *I* > 2σ(*I*)
*R*_int_ = 0.048

#### Refinement

*R*[*F* ^2^ > 2σ(*F* ^2^)] = 0.039
*wR*(*F* ^2^) = 0.116
*S* = 1.06
3041 reflections
177 parameters
H atoms treated by a mixture of independent and constrained refinement
Δρ_max_ = 0.30 e Å^−3^
Δρ_min_ = −0.24 e Å^−3^

### 

Data collection: *APEX2* (Bruker, 2005 ▶); cell refinement: *SAINT-Plus* (Bruker, 2004 ▶); data reduction: *SAINT-Plus* and *XPREP* (Bruker 2004 ▶); program(s) used to solve structure: *SHELXS97* (Sheldrick, 2008 ▶); program(s) used to refine structure: *SHELXL97* (Sheldrick, 2008 ▶); molecular graphics: *ORTEP-3 for Windows* (Farrugia, 1997 ▶) and *DIAMOND* (Brandenburg, 1999 ▶); software used to prepare material for publication: *WinGX* (Farrugia, 1999 ▶) and *PLATON* (Spek, 2009 ▶).

## Supplementary Material

Crystal structure: contains datablock(s) global, I. DOI: 10.1107/S1600536812032035/tk5129sup1.cif

Structure factors: contains datablock(s) I. DOI: 10.1107/S1600536812032035/tk5129Isup2.hkl

Supplementary material file. DOI: 10.1107/S1600536812032035/tk5129Isup3.cml

Additional supplementary materials:  crystallographic information; 3D view; checkCIF report

## Figures and Tables

**Table 1:** Hydrogen-bond geometry (Å, °)

*D*—H⋯*A*	*D*—H	H⋯*A*	*D*⋯*A*	*D*—H⋯*A*
N1—H1⋯O1	0.897 (16)	1.998 (15)	2.7041 (11)	134.6 (13)
N1—H1⋯O1^i^	0.897 (16)	2.392 (15)	3.0303 (12)	128.2 (12)

Symmetry code: (i) 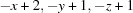.

**Fig. 1.**
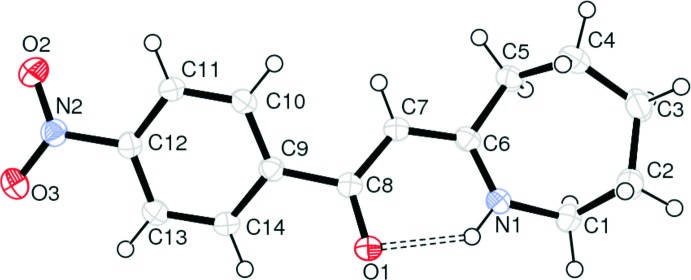
The molecular structure of (I) showing the atomic numbering scheme. Displacement ellipsoids are shown at the 50% probability level.

**Fig. 2.**
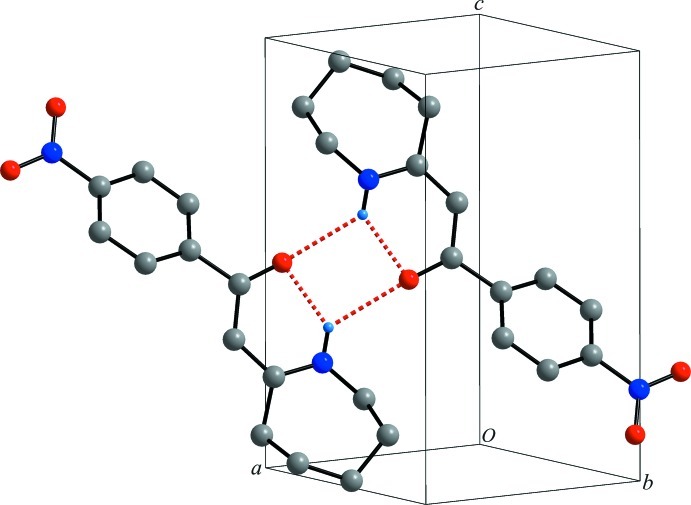
Hydrogen bonding diagram of the compound. Intermolecular and intramolecular N—H···O hydrogen bonds (shown as dashed red lines) form a four-membered ring. Non-participating H atoms have been omitted for clarity.

## supplementary crystallographic information

### Comment

The title compound was prepared as part of an ongoing methodological investigation into the use of enaminones in the synthesis of azabicyclic alkaloids (Michael *et al.*, 1999). For example, its reaction with acryloyl chloride, according to the method of Paulvannan & Stille (1994), produced a 2,3,6,7-tetrahydro-5(1*H*)-indolizinone related to numerous natural products. The crystal structures of analogous 4-bromophenyl enaminones with 5-, 6- and 7-membered rings have been reported (Balderson *et al.*, 2007).

The asymmetric unit of (I) consists of one molecule of 2-[(2*Z*)-azepan-2-ylidene]-1-(4-nitrophenyl)ethan-1-one (Fig. 1). The hydrogen bonding consists of an intramolecular N—H···O═C hydrogen bond, and an intermolecular N—H···O═C hydrogen bond (Table 1). The combination of these two hydrogen bonds results in an *R*^2^_2_(4) ring (Fig. 2) as described by graph set notation (Bernstein *et al.*, 1995).

### Experimental

The employed synthesis followed the Eschenmoser procedure (Roth *et al.*, 1971). *p*-Nitrophenacyl bromide (995 mg, 4.08 mmol) was added to a solution of azepane-2-thione (502 mg, 3.88 mmol) in dry acetonitrile (30 ml). The resulting solution was stirred at room temperature for 4 h, after which *S*-alkylation was complete as shown by the precipitation of the thioiminium salt. This was then followed by the addition of triphenylphosphine (1.069 g, 4.08 mmol) and triethylamine (413 mg, 4.08 mmol) to induce sulfur extrusion. The reaction mixture was poured into water and the organic components were extracted with diethyl ether (3 × 30 ml). The resulting organic layer was dried over MgSO_4_, filtered and the solvent removed *in vacuo*. The resulting residue was purified by column chromatography on silica gel with hexane:ethyl acetate (19:1 *v*/*v*) as eluent to yield 2-[(2*Z*)-azepan-2-ylidene]-1-(4-nitrophenyl)ethan-1-one (829 mg, 82%) as yellow crystals, m.p. 398–400 K.

### Refinement

The C-bound H atoms were geometrically placed [C—H = 0.95 Å (alkenyl- and aromatic-H) and 0.99 Å (methylene-H)] and refined as riding with *U*_iso_(H) = 1.2*U*_eq_(C). The N-bound H atom was located in a difference Fourier map and refined freely.

### Crystal data

**Table:**

C_14_H_16_N_2_O_3_	*Z* = 2
*M**_r_* = 260.29	*F*(000) = 276
Triclinic, *P*1	*D*_x_ = 1.362 Mg m^−^^3^
Hall symbol: -P 1	Mo *K*α radiation, λ = 0.71073 Å
*a* = 6.7963 (3) Å	Cell parameters from 6539 reflections
*b* = 8.4054 (3) Å	θ = 2.5–28.4°
*c* = 11.6649 (5) Å	µ = 0.10 mm^−^^1^
α = 76.508 (2)°	*T* = 173 K
β = 81.134 (2)°	Block, red
γ = 80.596 (2)°	0.56 × 0.5 × 0.42 mm
*V* = 634.58 (5) Å^3^	

### Data collection

**Table:**

Bruker APEXII CCD area-detector diffractometer	2732 reflections with *I* > 2σ(*I*)
ω scans	*R*_int_ = 0.048
Absorption correction: multi-scan (*SADABS*; Sheldrick, 1996)	θ_max_ = 28.0°, θ_min_ = 1.8°
*T*_min_ = 0.905, *T*_max_ = 0.955	*h* = −8→8
10354 measured reflections	*k* = −11→11
3041 independent reflections	*l* = −15→15

### Refinement

**Table:**

Refinement on *F*^2^	H atoms treated by a mixture of independent and constrained refinement
Least-squares matrix: full	*w* = 1/[σ^2^(*F*_o_^2^) + (0.0618*P*)^2^ + 0.1127*P*] where *P* = (*F*_o_^2^ + 2*F*_c_^2^)/3
*R*[*F*^2^ > 2σ(*F*^2^)] = 0.039	(Δ/σ)_max_ < 0.001
*wR*(*F*^2^) = 0.116	Δρ_max_ = 0.30 e Å^−^^3^
*S* = 1.06	Δρ_min_ = −0.24 e Å^−^^3^
3041 reflections	Extinction correction: *SHELXL97* (Sheldrick, 2008), Fc^*^=kFc[1+0.001xFc^2^λ^3^/sin(2θ)]^-1/4^
177 parameters	Extinction coefficient: 0.170 (12)
0 restraints	

### Special details

**Table:**

Experimental. Absorption corrections were made using the program *SADABS* (Sheldrick, 1996)
Geometry. All e.s.d.'s (except the e.s.d. in the dihedral angle between two l.s. planes) are estimated using the full covariance matrix. The cell e.s.d.'s are taken into account individually in the estimation of e.s.d.'s in distances, angles and torsion angles; correlations between e.s.d.'s in cell parameters are only used when they are defined by crystal symmetry. An approximate (isotropic) treatment of cell e.s.d.'s is used for estimating e.s.d.'s involving l.s. planes.

### Fractional atomic coordinates and isotropic or equivalent isotropic displacement parameters (Å^2^)

**Table:**

	*x*	*y*	*z*	*U*_iso_*/*U*_eq_	
C1	1.01720 (16)	0.40728 (13)	0.79045 (9)	0.0296 (2)	
H1A	1.0007	0.4794	0.8483	0.035*	
H1B	1.155	0.4094	0.7482	0.035*	
C2	0.99594 (18)	0.23125 (14)	0.85852 (10)	0.0342 (3)	
H2A	1.1187	0.1856	0.8974	0.041*	
H2B	0.9882	0.1633	0.8009	0.041*	
C3	0.8137 (2)	0.21416 (15)	0.95278 (11)	0.0394 (3)	
H3A	0.8141	0.096	0.9913	0.047*	
H3B	0.8279	0.2735	1.0144	0.047*	
C4	0.61162 (18)	0.27927 (13)	0.90694 (10)	0.0333 (3)	
H4A	0.5034	0.2527	0.9728	0.04*	
H4B	0.5976	0.2215	0.8443	0.04*	
C5	0.58338 (16)	0.46611 (13)	0.85608 (9)	0.0281 (2)	
H5A	0.4378	0.5065	0.8606	0.034*	
H5B	0.6428	0.5213	0.9062	0.034*	
C6	0.67677 (15)	0.51517 (12)	0.72942 (9)	0.0248 (2)	
C7	0.55481 (15)	0.60086 (12)	0.64327 (9)	0.0256 (2)	
H7	0.4135	0.6146	0.665	0.031*	
C8	0.63077 (15)	0.66855 (12)	0.52480 (9)	0.0249 (2)	
C9	0.48836 (15)	0.78776 (12)	0.44815 (8)	0.0236 (2)	
C10	0.28080 (15)	0.78647 (13)	0.46714 (9)	0.0267 (2)	
H10	0.2248	0.7046	0.5285	0.032*	
C11	0.15532 (15)	0.90342 (13)	0.39730 (9)	0.0270 (2)	
H11	0.0138	0.9038	0.411	0.032*	
C12	0.24127 (15)	1.01984 (12)	0.30693 (9)	0.0244 (2)	
C13	0.44670 (16)	1.02284 (12)	0.28387 (9)	0.0271 (2)	
H13	0.5021	1.1028	0.2207	0.033*	
C14	0.56934 (15)	0.90611 (13)	0.35534 (9)	0.0273 (2)	
H14	0.7107	0.9064	0.3411	0.033*	
N1	0.87397 (13)	0.47603 (11)	0.70420 (8)	0.0275 (2)	
H1	0.923 (2)	0.5042 (18)	0.6274 (14)	0.042 (4)*	
N2	0.10920 (13)	1.14105 (10)	0.23068 (8)	0.0275 (2)	
O1	0.81102 (11)	0.64279 (10)	0.48208 (7)	0.0328 (2)	
O2	−0.07169 (12)	1.15862 (11)	0.26345 (8)	0.0403 (2)	
O3	0.18635 (12)	1.21881 (10)	0.13628 (7)	0.0373 (2)	

### Atomic displacement parameters (Å^2^)

**Table:**

	*U*^11^	*U*^22^	*U*^33^	*U*^12^	*U*^13^	*U*^23^
C1	0.0269 (5)	0.0327 (5)	0.0286 (5)	−0.0022 (4)	−0.0067 (4)	−0.0047 (4)
C2	0.0361 (6)	0.0315 (5)	0.0320 (6)	0.0020 (4)	−0.0066 (5)	−0.0036 (4)
C3	0.0462 (7)	0.0339 (6)	0.0301 (6)	0.0000 (5)	−0.0018 (5)	0.0034 (4)
C4	0.0379 (6)	0.0319 (5)	0.0276 (5)	−0.0090 (4)	0.0047 (4)	−0.0041 (4)
C5	0.0294 (5)	0.0320 (5)	0.0213 (5)	−0.0027 (4)	0.0015 (4)	−0.0062 (4)
C6	0.0267 (5)	0.0249 (5)	0.0224 (5)	−0.0042 (4)	−0.0002 (4)	−0.0056 (4)
C7	0.0225 (5)	0.0283 (5)	0.0241 (5)	−0.0023 (4)	0.0005 (4)	−0.0045 (4)
C8	0.0238 (5)	0.0262 (5)	0.0241 (5)	−0.0030 (4)	−0.0017 (4)	−0.0050 (4)
C9	0.0241 (5)	0.0245 (5)	0.0218 (5)	−0.0030 (4)	−0.0008 (4)	−0.0057 (4)
C10	0.0265 (5)	0.0286 (5)	0.0233 (5)	−0.0064 (4)	−0.0012 (4)	−0.0017 (4)
C11	0.0213 (5)	0.0326 (5)	0.0262 (5)	−0.0048 (4)	−0.0016 (4)	−0.0047 (4)
C12	0.0265 (5)	0.0233 (5)	0.0235 (5)	−0.0016 (4)	−0.0040 (4)	−0.0055 (4)
C13	0.0283 (5)	0.0258 (5)	0.0257 (5)	−0.0063 (4)	−0.0014 (4)	−0.0020 (4)
C14	0.0217 (5)	0.0313 (5)	0.0272 (5)	−0.0050 (4)	−0.0006 (4)	−0.0036 (4)
N1	0.0253 (4)	0.0332 (5)	0.0211 (4)	−0.0019 (3)	−0.0017 (3)	−0.0023 (3)
N2	0.0282 (5)	0.0251 (4)	0.0286 (4)	−0.0026 (3)	−0.0040 (3)	−0.0047 (3)
O1	0.0236 (4)	0.0398 (4)	0.0278 (4)	0.0009 (3)	0.0021 (3)	0.0002 (3)
O2	0.0263 (4)	0.0435 (5)	0.0434 (5)	0.0029 (3)	−0.0038 (3)	0.0004 (4)
O3	0.0378 (5)	0.0363 (4)	0.0319 (4)	−0.0056 (3)	−0.0043 (3)	0.0049 (3)

### Geometric parameters (Å, º)

**Table:**

C1—N1	1.4630 (13)	C7—C8	1.4174 (14)
C1—C2	1.5254 (15)	C7—H7	0.95
C1—H1A	0.99	C8—O1	1.2535 (12)
C1—H1B	0.99	C8—C9	1.5073 (14)
C2—C3	1.5259 (17)	C9—C10	1.3952 (14)
C2—H2A	0.99	C9—C14	1.3990 (14)
C2—H2B	0.99	C10—C11	1.3870 (14)
C3—C4	1.5219 (18)	C10—H10	0.95
C3—H3A	0.99	C11—C12	1.3877 (14)
C3—H3B	0.99	C11—H11	0.95
C4—C5	1.5353 (15)	C12—C13	1.3838 (14)
C4—H4A	0.99	C12—N2	1.4694 (13)
C4—H4B	0.99	C13—C14	1.3848 (15)
C5—C6	1.5060 (13)	C13—H13	0.95
C5—H5A	0.99	C14—H14	0.95
C5—H5B	0.99	N1—H1	0.897 (16)
C6—N1	1.3306 (13)	N2—O2	1.2253 (12)
C6—C7	1.3927 (14)	N2—O3	1.2300 (12)
			
N1—C1—C2	114.74 (9)	C7—C6—C5	119.10 (9)
N1—C1—H1A	108.6	C6—C7—C8	123.35 (9)
C2—C1—H1A	108.6	C6—C7—H7	118.3
N1—C1—H1B	108.6	C8—C7—H7	118.3
C2—C1—H1B	108.6	O1—C8—C7	124.12 (9)
H1A—C1—H1B	107.6	O1—C8—C9	118.04 (9)
C1—C2—C3	115.04 (9)	C7—C8—C9	117.72 (9)
C1—C2—H2A	108.5	C10—C9—C14	119.09 (9)
C3—C2—H2A	108.5	C10—C9—C8	122.91 (9)
C1—C2—H2B	108.5	C14—C9—C8	117.99 (9)
C3—C2—H2B	108.5	C11—C10—C9	120.69 (9)
H2A—C2—H2B	107.5	C11—C10—H10	119.7
C4—C3—C2	115.06 (10)	C9—C10—H10	119.7
C4—C3—H3A	108.5	C10—C11—C12	118.47 (9)
C2—C3—H3A	108.5	C10—C11—H11	120.8
C4—C3—H3B	108.5	C12—C11—H11	120.8
C2—C3—H3B	108.5	C13—C12—C11	122.46 (9)
H3A—C3—H3B	107.5	C13—C12—N2	118.91 (9)
C3—C4—C5	113.73 (9)	C11—C12—N2	118.60 (9)
C3—C4—H4A	108.8	C12—C13—C14	118.20 (9)
C5—C4—H4A	108.8	C12—C13—H13	120.9
C3—C4—H4B	108.8	C14—C13—H13	120.9
C5—C4—H4B	108.8	C13—C14—C9	121.06 (9)
H4A—C4—H4B	107.7	C13—C14—H14	119.5
C6—C5—C4	113.97 (9)	C9—C14—H14	119.5
C6—C5—H5A	108.8	C6—N1—C1	126.07 (9)
C4—C5—H5A	108.8	C6—N1—H1	115.6 (9)
C6—C5—H5B	108.8	C1—N1—H1	118.0 (9)
C4—C5—H5B	108.8	O2—N2—O3	123.31 (9)
H5A—C5—H5B	107.7	O2—N2—C12	118.66 (9)
N1—C6—C7	122.38 (9)	O3—N2—C12	118.02 (9)
N1—C6—C5	118.52 (9)		
			
N1—C1—C2—C3	−73.34 (13)	C9—C10—C11—C12	1.08 (16)
C1—C2—C3—C4	58.23 (14)	C10—C11—C12—C13	0.29 (16)
C2—C3—C4—C5	−64.07 (13)	C10—C11—C12—N2	178.33 (9)
C3—C4—C5—C6	82.62 (12)	C11—C12—C13—C14	−0.94 (16)
C4—C5—C6—N1	−60.10 (13)	N2—C12—C13—C14	−178.98 (9)
C4—C5—C6—C7	120.49 (11)	C12—C13—C14—C9	0.24 (16)
N1—C6—C7—C8	−6.73 (16)	C10—C9—C14—C13	1.07 (16)
C5—C6—C7—C8	172.66 (10)	C8—C9—C14—C13	−177.89 (9)
C6—C7—C8—O1	8.38 (17)	C7—C6—N1—C1	170.95 (10)
C6—C7—C8—C9	−167.41 (9)	C5—C6—N1—C1	−8.44 (15)
O1—C8—C9—C10	157.98 (10)	C2—C1—N1—C6	68.52 (14)
C7—C8—C9—C10	−25.96 (14)	C13—C12—N2—O2	−166.71 (9)
O1—C8—C9—C14	−23.10 (14)	C11—C12—N2—O2	15.17 (14)
C7—C8—C9—C14	152.95 (10)	C13—C12—N2—O3	13.97 (14)
C14—C9—C10—C11	−1.74 (16)	C11—C12—N2—O3	−164.14 (9)
C8—C9—C10—C11	177.16 (9)		

### Hydrogen-bond geometry (Å, º)

**Table:**

*D*—H···*A*	*D*—H	H···*A*	*D*···*A*	*D*—H···*A*
N1—H1···O1	0.897 (16)	1.998 (15)	2.7041 (11)	134.6 (13)
N1—H1···O1^i^	0.897 (16)	2.392 (15)	3.0303 (12)	128.2 (12)

Symmetry code: (i) −*x*+2, −*y*+1, −*z*+1.
